# Price guidance and discovery of the Chinese stock index Futures: Based on the rising, falling and fluctuating states

**DOI:** 10.1016/j.heliyon.2023.e14429

**Published:** 2023-03-09

**Authors:** Min Su

**Affiliations:** College of Economics and Management, Taiyuan University of Technology, Taiyuan, China

**Keywords:** Stock index futures, Price discovery, Fluctuating states, Steady market

## Abstract

Stock index futures have been around for more than 12 years in the Chinese market, but there are conflicting viewpoints on the role of Chinese stock index futures in the market. Many crises, including COVID-19, have heightened the financial system's vulnerability and instability. Do China's stock index futures play a role in stabilizing the market and discovering prices in turbulent times? This study employs a combination of VEC, PT, and IS methodologies to investigate the lead-lag relation and price discovery ability of stock index futures markets. By evaluating price interactions in three different scenarios, we reveal that stock index futures play a prominent role in price discovery, particularly in markets with excessive volatility. However, their impact on price discovery is weaker during stable market conditions. To the best of our knowledge, this study is the first to categorize the Chinese stock market into different states, providing valuable insights into the price discovery function of stock index futures. Our findings have important implications for policymakers and investors, as they highlight the need for increased attention to market manipulation and excessive speculation during volatile market conditions to protect the interests of investors.

## 1 Introduction

Stock index futures have been widely developed and issued since their beginning in the United States in 1982, becoming one of the world's most actively traded and liquid futures. China officially launched the CSI 300 futures on April 16, 2010, followed by the SSE 50 and CSI 500 futures. The launch of China's stock index futures marks a turning point in the capital market, signaling that investors have shorting mechanisms. Despite initial expectations of market stabilization and risk reduction, the stock market dropped by more than 30% just three months after the launch of stock index futures, causing investors to doubt its effectiveness. Nevertheless, some empirical studies suggest that stock index futures reduce volatility in China's markets [1,2]. The recent global economic crises, including the COVID-19 pandemic, have exacerbated the financial market's fragility and volatility [3,4]. In such chaotic times, do China's stock index futures play a role in mitigating risk and discovering prices?

Price discovery is one of the most significant functions of the stock index futures market. Most of the literature has shown that informed traders often choose to trade in a low-cost futures market [[5], [6], [7], [8]], and changes in futures prices often lead to changes in spot prices, a phenomenon that has been extensively studied in the literature. Schreiber & Schwartz [9] found that price discovery is a process of quickly and effectively integrating market-related information into asset prices and finding equilibrium prices. Chan [10] provided statistically significant evidence that the S&P 500 futures market leads the spot market. Fleming et al. [11] argued that informed traders usually choose to trade in a low-cost futures market, so futures prices will be the first to absorb and reflect those innovations. Kim [12] employed a VAR model to explore the futures and spot markets of the S&P500, MMI, and NYSE composite and found that the S&P 500 index had a leading and dominant position in the futures market, while the MMI index had a similar position in the spot market.

However, not all studies have found a clear relationship between futures and spot prices. Some studies have found that the spot market index price dominates the futures index [[13], [14], [15]], while others have found a bidirectional causal relationship between the two markets. Pizzi et al. [16] discovered that the stock index and its associated futures index could lead to each other differently depending on the situation. Ausloos et al. [17] used TGARCH methodologies to investigate the influence of CSI-300 index futures trading on spot price volatility and found a stationary equilibrium and a bidirectional Granger causality between the CSI-300 spot and futures markets and the introduction of CSI-300-IF considerably reduced volatility.

The inconsistency mentioned above may be inherent in the very nature of heterogeneity. The price discovery function may also differ dramatically among different countries [18,19] due to the differences in the trading volume, breadth, and depth of stock index futures markets in various countries [20,21]. For instance, Xiao and Wu [22] found that informed traders tend to trade in the stock market when the information reflects the operating conditions of a limited number of companies and in the futures market when the information reflects the overall market position. Similarly, Yang et al. [23] used agent-based modeling to build a theoretical model of the order book and discovered that speculators, arbitrageurs, and hedgers play distinct roles in Chinese markets. Furthermore, Zhou & Li [24] constructed a multiagent spot-futures market model to examine the micromechanics of shock transfer and observed that arbitrageurs play a substantial role in spot-futures market contact and that appropriate arbitrage trading during crises can aid in reducing the positive basis and preventing the further spread of crises.

In addition to the qualitative study on the price discovery mechanism, the existing literature has adopted a series of econometric instruments. These methodologies have evolved from simple linear regression to more advanced techniques, such as cointegration analysis, VECM, and time-varying algorithms. For example, Liu et al. [25] use the thermal optimal path approach to analyze the dynamics of the lead-lag relationship between the Chinese stock index and the futures market and find that the relationship changed significantly before and after the outbreak of COVID-19. Similarly, Ren et al. [26] use the thermal optimal route approach to study the SSE 50 Index and its derivatives and find that the stock index leads the index future, while the index option leads the index future. Lu et al. [27] investigate the link between WTI crude oil futures and S&P 500 index futures or CSI 300 futures and find that the mean link between them is diminishing.

China is the world's largest developing country and second-largest economy, with a rapidly growing futures market. In 2021, the trading volume of China's futures market reached 7.514 billion lots, accounting for approximately 12% of the global futures market. In particular, the trading volume of stock index futures in China was 66.74 million lots, experiencing a 2% increase. As China's power in the financial market continues to grow, the impact of its stock index futures on the global financial market also increases. However, China's capital market still faces several challenges, including a lack of transparency, openness, and derivatives, which limit its market functions and the use of risk management instruments. The dominance of retail investors in the Chinese financial market also sets it apart from developed countries. Research suggests that futures can enhance the volatility of the underlying spot market in an immature market, as seen with individual Chinese investors accounting for almost 70% of overall investment [28]. When these markets face panic, they tend to cause extreme volatility, with overreactions particularly severe in stocks with low institutional ownership.

After stock index futures were introduced in China, regulators and investors were concerned about their role in the capital market. The question remains whether these futures are tools for market manipulation and speculation or if they defend the interests of investors. To foster the growth of futures market, Chinese regulators introduced a series of policies. Studies have examined the effects of these policies on the market, such as the improvement of market stability and the increase in volatility. He et al. [1] found that after implementing new market regulations in 2015, the futures market in China became more sensitive to new information and capable of price discovery. However, this function weakened in the long run due to the lack of liquidity. Lin & Wang [2] utilized variance ratio and spectral shape tests to examine the effect of tightened trading rules on the market efficiency and price discovery function of the Chinese stock index futures in 2015 and found that these rules improved the market rather than worsening it. Xu et al. [29] applied the quantile regression approach to investigate the heterogeneous impact of liquidity on volatility in the Chinese stock index futures and found that illiquidity significantly increases volatility at the right tail. Chen & Shi [30] used the LVaR model to examine the relationship between high-frequency trading (HFT) and exogenous liquidity risk and found that HFT improves the return of the liquidity provider and reduces the exogenous liquidity risk significantly.

The above literature reveals conflicts and inconsistencies in the conclusions of the research on China stock index futures prices. Some scholars argue that China stock index futures have already demonstrated the ability of price discovery, while others hold a differing view. Moreover, limited attention has been given to examining the price relationship and discovery capabilities under diverse market conditions. In light of this, this study employs VEC, PT, and IS methodologies to examine the price relationship and discovery capabilities of stock index futures in China under various market conditions. We classify the market into three distinct situations, namely rising, declining, and fluctuating, and analyze the lead-lag relationship between stock index futures and spot markets under each classification. This approach contributes to the existing literature by providing a novel perspective on the pricing relationship. Given that the demand for index futures fluctuates based on market conditions, it is important to examine the role of stock index futures under various market circumstances. In some cases, there is a significant demand for risk management, while in others, there is a limited need. The rapid increase and fall of stock prices in China have led to concerns over futures speculation, particularly in an immature market. Although the primary purpose of stock index futures is to hedge risks, instances of manipulation and speculation have been identified in the trading process.

Our findings reveal that stock index futures play a substantial role in price discovery during market upswings and downswings but have a limited impact during market stability. In conclusion, this study highlights the importance of policymakers and investors paying closer attention to the role that stock index futures play in the stock market's rapid rise and collapse. In order to enhance the development of the futures market in China, policymakers should expedite the growth of the futures market, increase the supply of financial derivatives, broaden the depth and breadth of financial futures, and strengthen the market mechanism.

The remainder of this paper is structured as follows: In Section 2, we present the data and outline the methodology employed. The empirical results are presented and analyzed in Section 3. To further validate our findings, robustness tests are conducted in Section 4. Finally, in Section 5, we provide the conclusions and implications of our research.

## 2 Data and methodology

### 2.1 Data

In this study, we examine the CSI 300 stock index, which represents the typical Chinese stock market and is comprised of 300 weighted stocks from both the Shanghai and Shenzhen markets. High-frequency data is widely favored by researchers in this field [31]. Therefore, our research object is the 1-min continuous index created by CSI 300 IF. As the trading hours for futures and spot markets differ, we only consider the CSI 300 IF data within the hours of 9:31–11:30 a.m. and 1:01–3:00 p.m., resulting in 240 1-min data points per trading day. The data is sourced from the wind database. To address the issue of heteroscedasticity, we apply natural logarithm processing on both the futures and spot sequences, which are denoted as LNIF and LNHS, respectively. In terms of sample selection, we choose three representative time periods for analysis:

The first phase is the stock market's remarkable ascent (Jan 4, 2019–Mar 6, 2019), during which the CSI300 rose from 3036 to 3848 points in just 39 trading days, reflecting a gain of 26.8% with an average daily increase of 0.7%.

The second phase is the rapid decline of the stock market (April 22, 2019–May 23, 2019), during which the CSI300 dropped from 4026 to 3584 points, or a decline of 11%, over the course of 21 trading days, with a daily average decrease of −0.5%.

The third phase is the stock market's slight fluctuation (November 1, 2018–November 28, 2018), during which the stock index remained relatively stable, fluctuating from 3174 to 3171, a change of just 0.001% over 20 trading days.

Table 1 represents the descriptive statistics of the data. From the skewness, kurtosis, and J-B statistics of the data in the three periods, we can judge that these data reject normal distribution and have the characteristics of sharp peaks and thick tails of general financial series.

**Table 1:** Descriptive statistics of prices of stock index futures and spot series.

	Rising phase		Declining phase		Fluctuating phase	
	LNIF	LNHS	LNIF	LNHS	LNIF	LNHS
N. of Obs.	9360	9360	5520	5520	4800	4800
Trading days	39	39	23	23	20	20
Mean	3334	3329	3742	3752	3214	3213
Amplitude	26.1%	25.8%	−11.1%	−10.3%	0.01%	−0.02%
Max	3847	3848	4025	4030	3294	3306
Min	3041	3036	3558	3584	3137	3126
Std. Dev.	255	251	158	150	46	50
Skewness	0.0448	0.0165	−0.2673	−0.2354	−0.4529	−0.3530
Kurtosis	1.9392	1.9265	1.7361	1.6747	2.9101	2.4291
Jarque-Bera	407.9***	415.3***	414.3***	435.2***	414.3***	412.5***

Note: *** indicates rejection of the null hypothesis at 1% significance level. J-B statistic is a test for normality.

### 2.2 Vector error correction model (**VECM**) analysis

We denote the two I (1) processes of futures and spot price time-series as the following vector error correction model (VECM) forms:

(1) $${\Delta LNIF}_{t}=\lambda_{f}e_{t-1}+\sum_{i=1}^{p}a_{fi}\Delta \ln\;{HS}_{t-i}+\sum_{i=1}^{p}b_{fi}\Delta \ln\;{IF}_{t-i}+\varepsilon_{f,t}$$

(2) $${\Delta LNHS}_{t}=\lambda_{s}e_{t-1}+\sum_{i=1}^{p}a_{si}\Delta \ln\;{HS}_{t-i}+\sum_{i=1}^{p}b_{si}\Delta \ln\;{IF}_{t-i}+\varepsilon_{s,t}\;$$

Where Δ is the first-order difference symbol, $e_{t-1}$ is the error correction term, $\lambda_{f}$ and $\lambda_{s}$ are the adjustment coefficients for the futures and spot error correction items respectively, $a_{fi}$、 $b_{fi}$、 $a_{si}$、 $b_{si}$ are short-term coefficients, p is the order of the equation, $\varepsilon_{f,t}$ and $\varepsilon_{s,t}$ are the residual terms. The VECM could capture the dynamic relationship between futures and spot prices from both long-term and short-term perspectives, and the cointegrating relationship describes the long-run equilibrium.

From the perspective of adjusting price series to the long-run equilibrium, the coefficients $\lambda_{f}$ and $\lambda_{s}$ of the Error Correction Model (ECM) can take one of three values: (1) If $\lambda_{f}<0$ and $\lambda_{s}>0$, the model is considered realistic and suitable. In this case, deviations from long-run equilibrium in futures and spot prices trigger a callback mechanism, with a faster callback resulting in a larger coefficient value. (2) Conversely, if $\lambda_{f}>0$ and $\lambda_{s}<0$, no callback mechanism is activated, rendering the prospect of long-term cointegration unlikely. (3) Finally, if $\lambda_{f}=0$, the outcome for $\lambda_{s}$ can be positive, zero, or negative. When $\lambda_{s}>0$, the spot market is driven towards the futures market, even if the latter is not constrained by the equilibrium relationship. If $\lambda_{s}$ = 0 or $\lambda_{s}$ <0, however, the cointegration relationship is negated, indicating that short-term changes are not restricted by long-term correlations.

In terms of a short-term relationship, if the coefficients $a_{fi}$ of $\Delta \ln\;{HS}_{t-i}$ in Equation (1) are found to be significantly non-zero, it implies that the spot price in prior periods has a significant effect on the futures price. The broader the period range considered, the sooner one can predict the futures price. Likewise, if the coefficients $b_{si}$ of $\Delta \ln\;{IF}_{t-i}$ in Equation (2) are significantly non-zero, it indicates that the futures price has a significant impact on the spot price. The longer the period span considered, the more accurate the futures price becomes as a forecast for the spot price, suggesting that the futures price tends to be ahead of the current spot price.

### 2.3 Common factor model

The Vector Error Correction Model (VECM) provides valuable information on the two markets' mutual guiding direction and leader-lag connection. However, it is crucial to determine the extent of each market's contribution to price discovery. To address this issue, two models have been introduced. The first model, Information Share (IS), introduced by Hasbrouck [7], decomposes the cofactor variance, while the second model, Permanent Transitory (PT), introduced by Gonzalo-Granger [32], decomposes the cofactors. The results of these two models may diverge significantly when residuals of the two equations are sufficiently correlated, while they may only differ slightly when the residuals are uncorrelated, as observed by Baillie [33].

#### 2.3.1 Permanent Transitory (P-T) model

Stock and Watson [34] introduced a cointegration model for futures and spot prices, $Y_{t}$, which was defined as the sum of a common factor, $F_{t}$, and a temporary component, $G_{t}$. Specifically, $Y_{t}=F_{t}+G_{t}$. Further research by Gonzalo and Granger [32] explored the common factor, $F_{t}$, and showed that it could be expressed as a linear combination of two prices. Specifically, $F_{t}=\beta Y_{t}$, where β is a vector of coefficients for the common factors. Given that β and the error correction coefficients vector, α, are orthogonal, the contribution of each market price can be calculated by standardizing β and expressing it as a linear combination, as shown in Eq. (3):

(3) $$\beta_{1}=\frac{\left|\alpha_{2}\right|}{\left|\alpha_{1}\right|+\left|\alpha_{2}\right|}\;\beta_{2}=\frac{\left|\alpha_{1}\right|}{\left|\alpha_{1}\right|+\left|\alpha_{2}\right|}$$

#### 2.3.2 Information share (I–S) model

By calculating the contribution of the variance, Hasbrouck [35] decomposed the variance of common factors and estimated the price discovery function of each market. When the new information in the two markets is highly uncorrelated, Eq. (4) indicates the information share of the I-th market:

(4) $$s_{i}=\frac{\gamma_{i}^{2}\sigma_{i}^{2}}{\sum_{i=1}^{n}\gamma_{i}^{2}\sigma_{i}^{2}}$$

We could apply the Cholesky decomposition technique when there is a correlation between innovations in two markets. Nonetheless, the order of the variables affects this strategy. As a result, by adjusting the order of the two variables, we can get the upper and lower limits of each market IS, and the results are shown in Eq. (5) as follows:

(5) $${IS}_{F}^{U}=\frac{{\left(\lambda_{s}\sigma_{f}-\lambda_{f}\rho \sigma_{s}\right)}^{2}}{{\lambda_{s}}^{2}{\sigma_{f}}^{2}-2\lambda_{f}\lambda_{s}\rho \sigma_{f}\sigma_{s}+{\lambda_{f}}^{2}{\sigma_{s}}^{2}}\;{IS}_{F}^{L}=\frac{{\lambda_{s}}^{2}{\sigma_{f}}^{2}{(1-\rho }^{2})}{{\lambda_{f}}^{2}{\sigma_{s}}^{2}-2\lambda_{f}\lambda_{s}\rho \sigma_{f}\sigma_{s}+{\lambda_{s}}^{2}{\sigma_{f}}^{2}}$$

In this study, we use the average of ${IS}_{F}^{U}$ and ${IS}_{F}^{L}$ in Eq. (5) as reliable estimates of the futures market share in price discovery. Similarly, we may employ this strategy to estimate the spot market.

## 3 Results and discussion

### 3.1 Unit root and cointegration test

In examining the interaction relationship between the two markets, it is important to assess the presence of a stable co-integrating link in the long run. To this end, we perform a stationarity and cointegration analysis. The Augmented Dickey-Fuller (ADF) test is conducted as the first step in this process. The results reveal that the level series is not stationary at the 1% significance level. However, upon performing the first difference, the series becomes stationary, as shown in Table 2. This result indicates that the two series are I (1) processes, which allows us to proceed with the cointegration analysis.

**Table 2:** ADF test results of CSI300 futures and spot.

	Rising phase	Declining phase	Fluctuating phase
LNIF	−4.06	−3.69	−2.92
ΔLNIF	−94.07***	−73.14***	−116.43***
LNHS	−1.32	−3.25	−3.01
ΔLNHS	−40.56***	−38.61***	−53.73***

Note: *** indicates rejection of the null hypothesis at 1% significance level.

We adopt the Trace Statistic proposed by Johansen and Juselius [36] to assess the cointegrating relationship, as the E-G test has been criticized in the literature for several faults. Our approach assumes that the series contain trend terms, while the cointegration equation only includes constant components. The order of the VAR model is determined using the standard approach, which is the VAR model's order-minus-one criteria. The results, presented in Table 3, show that the assumption of non-cointegration in the first row is rejected at a 1% significance level. On the other hand, the second row indicates the existence of at least one cointegration connection, implying a long-term cointegration tendency between stock index futures and spot equities.

**Table 3:** Results of JJ cointegration relationship test between SS300 futures and spot market.

H0	Rising phase		Declining phase		Fluctuating phase	
	Eigenvalues	$\lambda_{trace}$	Eigenvalues	$\lambda_{trace}$	Eigenvalues	$\lambda_{trace}$
r = 0	0.006194	55.35*** (0.00)	0.005558	29.97*** (0.00)	0.003871	55.29*** (0.00)
r = 1	0.000197	1.70388 (0.19)	0.000109	0.5765 (0.48)	0.000731	1.5683 (0.25)

Note: *** indicates that the null hypothesis is rejected at the 1% significance level, and the combined probability is within brackets.

### 3.2 Vector error correction model (VECM) analysis

This section explores the price co-integration relationship under three conditions: rising, declining, and fluctuating.

Firstly, we analyze the stock market trend from January 4, 2019 to March 6, 2019, during which the five major moving averages, including the 5-day, 10-day, 20-day, and 60-day averages, showed an upward trend for 39 trading days. Utilizing the AIC and SC minimum criteria, the order of the VECM model was determined to be eight and all AR roots were found to be within the unit circle, indicating a stable model. The results of the rising phase, as presented in Table 4 (column of the rising phase), suggest that the constraints of the long-term equilibrium relationship have limited impact on the short-term fluctuations of futures returns. This is evidenced by the fact that the adjustment coefficient $\lambda_{f}$ of futures return is not significantly different from 0. In contrast, the adjustment coefficient $\lambda_{s}$ of the spot significantly differs from 0 at a significance level of 1%, with $\lambda_{s}$ > 0. These empirical findings imply that long-term linkages do not bind the futures market. On the other hand, the spot market is constrained by long-term equilibrium and will actively move closer to the futures market. From the perspective of short-term relationships, the spot market rate of return $({\Delta LNHS}_{t}$）is significantly affected by the futures market's first, second, third, fourth, fifth, and sixth periods (confidence 99%). The empirical findings suggest that the futures market can outperform the spot market by 6 min. However, in the futures yield equation, the spot market only has a significant influence of 0.092 in 1 min, while the influence is not significant for the rest of the time. To summarize, during periods of rising stock prices, the price discovery function of the futures market outperforms that of the spot market in both the long and short term.

**Table 4:** Estimation results of the three-phase VECM.

	Rising phase		Declining phase		Fluctuating phase	
	${\Delta LNIF}_{t}$	${\Delta LNHS}_{t}$	${\Delta LNIF}_{t}$	${\Delta LNHS}_{t}$	${\Delta LNIF}_{t}$	${\Delta LNHS}_{t}$
${Ecm}_{t-1}$	−0.0030[-0.91]	0.0086[ 3.60]	−0.0093[-2.19]	0.0064[2.12]	−0.0082[-4.23]	0.0023[1.77]
${\Delta LNIF}_{t-1}$	−0.057[-3.86]	0.207[19.83]	−0.023[-1.24]	0.221[16.94]	−0.146[-14.21]	0.143[21.08]
${\Delta LNIF}_{t-2}$	−0.018[-1.19]	0.122[11.17]	−0.024[-1.22]	0.111[8.11]	−0.098[-9.09]	0.080[11.33]
${\Delta LNIF}_{t-3}$	0.0001[0.003]	0.120[10.82]	−0.002[-0.10]	0.08[5.83]	−0.065[-6.00]	0.062[8.60]
${\Delta LNIF}_{t-4}$	0.0199[1.27]	0.109[9.77]	0.060[3.15]	0.086[6.31]	−0.043[-3.95]	0.056[7.73]
${\Delta LNIF}_{t-5}$	0.011[0.71]	0.063[5.67]	0.057[3.09]	0.074[5.67]	−0.041[-3.73]	0.035[4.86]
${\Delta LNIF}_{t-6}$	−0.001[-0.043]	0.035 [3.12]	–	–	0.003[ 0.25]	0.047[6.42]
${\Delta LNIF}_{t-7}$	−0.041[-2.67]	0.010[0.93]	–	–	−0.010[-0.94]	0.026[3.57]
${\Delta LNIF}_{t-8}$	−0.023[-1.62]	0.022[ 2.19]	–	–	−0.021[-2.00]	0.017[2.41]
${\Delta LNIF}_{t-9}$	–	–	–	–	−0.002[-0.17]	0.021[3.04]
${\Delta LNHS}_{t-1}$	0.092[ 4.50]	0.157[10.87]	0.039[ 1.55]	0.022[ 1.23]	0.24[15.53]	0.021[2.09]
${\Delta LNHS}_{t-2}$	−0.0195[-0.95]	−0.033[-2.24]	−0.009[-0.36]	−0.078[-4.29]	0.29[18.78]	0.026[2.50]
${\Delta LNHS}_{t-3}$	−0.004[-0.196]	−0.096[-6.63]	−0.032[-1.26]	−0.127[-7.06]	0.00[0.16]	−0.044[-4.20]
${\Delta LNHS}_{t-4}$	−0.041[-1.02]	−0.111[-7.81]	−0.068[-2.70]	−0.138[-7.74]	0.013[0.86]	−0.035[-3.37]
${\Delta LNHS}_{t-5}$	−0.013[-0.66]	−0.062[-4.35]	−0.008[-0.33]	−0.05[-3.08]	0.032[2.04]	−0.022[-2.13]
${\Delta LNHS}_{t-6}$	−0.016[-0.81]	−0.051[-3.58]	–	–	0.013[0.85]	−0.027[-2.62]
${\Delta LNHS}_{t-7}$	0.035[1.36]	−0.028[-2.01]	–	–	−0.024[-1.52]	−0.037[-3.55]
${\Delta LNHS}_{t-8}$	0.001[0.03]	−0.053[-4.2]	–	–	−0.026[-1.64]	−0.042[-4.14]
${\Delta LNHS}_{t-9}$	–	–	–	–	−0.008[-0.51]	−0.006[0.57]

Note: T-values in parenthesis, and the "------" means there is no such item.

Secondly, we choose the period (April 22 to May 23, 2019) as the dropping phase, a total of 23 trading days with an 11% decline. Using the AIC and SC minimum criterion, the order of the VECM was determined to be 5 and all the AR roots were found to be within the unit circle, indicating a stable model. The results of the empirical analysis during the declining phase are presented in the middle column of Table 4. The results suggest that there is a significant long-term relationship between the futures and spot returns during short-term adjustments, with a significance level of 5%. It was found that the adjustment factors of the futures and spot returns were negatively and positively correlated, respectively. Specifically, the futures return was found to be negative while the spot return was positive. However, the futures adjustment coefficient has a higher absolute value than the spot. Such findings imply that both the futures and spot markets are bound by long-term equilibrium and will move closer to one another, although the futures market will change more rapidly. From the short-term mutual relationship, the spot market return is affected by the futures market's first, second, third, fourth, and fifth periods, which are significantly non-zero. As a result, we can observe that the futures market price is 5 min ahead of the spot market price. In the equation of the future (Eq. (1)), the spot market only dominates for 4 min and is insignificant for the remainder of the period. As a result, the spot market has increased its price guidance and discovery function, but it is limited after all, and the futures market plays a leading role in this process.

Thirdly, we choose the period (Nov 1 to Nov 28, 2018) as the fluctuating phase, a total of 20 trading days. According to the above model selection criteria, the order of VECM is 9, and AR roots are all within the unit circle, indicating that the model is stable. The specific results are shown in Table 4 (the last column of fluctuating phase). From the perspective of long-term constraints on short-term, the adjustment coefficient of the spot yield is not significant at 1% and 5% levels but significant at 10%, and the adjustment coefficient of futures is significantly not equal to 0 and less than 0 at the 1% level. The absolute value of the futures adjustment coefficient is greater than the spot. These empirical findings imply that while the long-term equilibrium relationship also constrains the spot market, the futures market is more constrained by that relationship and will proactively shift toward the spot market. Therefore, in a period of stock market volatility, if we observe from a long-term perspective, we can find price guiding function of the spot market may outperform the future market.

### 3.3 Common factor model

Based on the VECM analysis, we employ the PT and IS methodologies to quantify the share of various markets in price determination. The specific results are reported in Table 5.

**Table 5:** IS and PT model test results.

Phase	*IS* model test results			*PT* model test results	
	Future market%	Spot market%	Correlation	Future market%	Spot market%
Rising	68.6% （97.5% 39.6%）	31.4% （2.5% 60.4%）	0.667	73.8%	26.2%
Declining	49.5% （82.2% 16.9%）	50.5% （17.8% 83.1%）	0.653	40.8%	59.2%
Fluctuating	28.8% （48.3% 9.28%）	71.2% （51.7% 90.7%）	0.443	21.9%	78.1%

Note: In parentheses, the preceding and following figures represent the upper and lower limits of information market share, respectively.

In the fast-rising phase (see the first row of Table 5), both the PT and IS techniques demonstrate that the futures index has a high proportion of price discovery (73.8% and 68.6%, respectively), which is much higher than the spot market (26.2% and 31.4%). In addition, the futures-spot correlation coefficient reached 0.667%, indicating a strong correlation, and the IS model is more reliable than the PT. This result is also consistent with our VECM findings, which indicate that the futures market has a much stronger price discovery ability than the spot market during the rapid rise period.

During the rapid fall phase (see the second row of Table 5), the IS model indicates that the share of futures and spot market price discovery is 49.5% and 50%, respectively, with no significant difference. According to the PT model, the spot market has an almost 60% impact. Nonetheless, given a correlation coefficient of 0.653, indicating a weak association between the two markets, we may conclude that the IS strategy produces more reliable results than the PT method. In other words, there is little difference in the share of price discovery power between the two markets.

When the stock market is turbulent (see the third row of Table 5), both the PT and IS models demonstrate that the spot market accounts for a considerable percentage of price discovery, accounting for 78.1% and 71.2%, respectively. In addition, as the correlation coefficient is 0.443, indicating a relatively weak correlation, we may conclude that the results of the two models have little difference. This result is also consistent with our previous VECM, which recognizes that the price discovery of the spot market outperforms that of the futures market under turbulent conditions.

## 4 Robustness test

In order to enhance the reliability and validity of the findings, robustness tests were conducted for two additional phases, consisting of six periods. The specific sample periods are detailed in Table 6.

**Table 6:** Two phases of stock index futures and spot sequence.

	Rising	Fluctuating	Declining
Phase II	2015.3.9–2015.4.28	2014.5.12–2014.7.21	2015.6.9–2015.7.9
Phase III	2015.5.8–2015.6.12	2016.3.24–2016.4.18	2015.12.25–2016.1.29

Using Phase II and III data, we performed IS and PT tests. The results show that the futures market's price discovery ability is much stronger than that of the spot market for both rising and falling prices (see Table 7), which is consistent with the previous non-robustness test results. In contrast, during periods of small volatility, the spot market slightly outperforms the futures market in price discovery, consistent with the non-robust stage results. The mean value of the two fluctuating phases of the robustness test is about 72.5% (see Table 7). Moreover, the explanation for this phenomenon is that the stronger price discovery ability in futures markets may be related to the greater sensitivity to bad news or the so-called higher tail risk in financial markets.

**Table 7:** IS and PT model test results for Phase 2 and Phase 3.

Phase		IS methods test results			PT methods test results	
		Future market%	Spot market%	Correlation	Future market%	Spot market%
Phase II	Rising	70.6% （95.5% 45.6%）	29.4% （4.5% 54.4%）	0.686	65.6%	34.4%
	Declining	72.6% （88.2% 56.9%）	27.4% （11.8% 43.1%）	0.753	71.5%	28.5%
	Fluctuating	34.8% （50.3% 19.3%）	65.2% （49.7% 80.7%）	0.321	24.9%	75.1%
Phase III	Rising	76.6% （93.6% 59.5%）	23.4% （6.4% 41.5%）	0.695	73.6%	26.4%
	Declining	79.1% （86.2% 72.1%）	20.9% （13.8% 17.9%）	0.781	75.1%	24.9%
	Fluctuating	36.5% （49.1% 23.9%）	63.5% （50.9% 76.1%）	0.401	30.4%	69.6%

Note: In parentheses, the preceding and following figures represent the upper and lower limits of information market share, respectively.

## 5 Conclusion and implications

Stock index futures are a relatively new financial derivative for China. In this study, we first divide the stock market into three distinct states: rising, falling, and fluctuating, and then utilize the VEC, PT, and IS methodologies to examine price discovery ability between stock index futures and spot markets. Our empirical results show the following: (1) When the stock market increases rapidly (in an up-trend), stock index futures are six periods ahead of the spot market due to their unique microstructure, which allows for rapid reaction and improved absorption of macroeconomic information, accounting for around 70% of price discovery. (2) During periods of fast decline (such as bear markets), stock index futures lead the spot market by about five periods, even if the fraction of price discovery is identical. It may be due to China's futures market's current small size and lack of diversity. (3) In the period of low volatility, the price discovery ability of futures is poorer than that of the spot market, accounting for only around 25%, which may be due to the lack of market hot spots or investor desire to use futures for arbitrage or hedging. In conclusion, stock index futures play a critical role during market upswings and downswings but less during market stability.

Accordingly, the above results and discussions have significant implications for policymaking and investment practice. Regulators and investors should pay closer attention to the role that stock index futures play in the rapid rise and fall of the stock market. Since when the stock market moves dramatically, it is an opportunity for some people to use stock index futures to engage in speculative arbitrage or market manipulation. Even though China's securities regulators have rarely addressed stock index futures manipulation in public, such purported events are occasionally reported by some media. The manipulation of stock index futures markets is regarded as a “non-indictable crime” because it is difficult to detect and regulate. As a result, regulators should pay particular attention to the movement of stock index futures and significantly strengthen supervision to prevent market manipulation from damaging the interests of small and medium-sized investors during periods of extreme stock market volatility.

Despite providing empirical evidence for developing the stock index futures, this study has several limitations. The first limitation is the availability of data. In this research, 9360 1-min data were used, but only for a 2-year period, which lacks representativeness at a broader scale. In future research, a more extended period and additional data levels should be used to test the validity of the results. Second, the impact mechanisms require extensive research and documentation to provide more actionable implementation advice. Third, China's stock index futures are undergoing dynamic and rapid development. The direction of future research should include some factors related to this dynamic transformation to shed light on price discovery of Chinese stock index futures.

## Author contribution statement

Min Su: Conceived and designed the analysis; Analyzed and interpreted the data; Examined and evaluated the findings; Wrote the paper.

## Funding statement

Prof. Min Su was supported by 10.13039/501100012456National Social Science Fund of China [21BJY125].

## Data availability statement

Data will be made available on request.

## Declaration of competing interest

The authors declare no competing interests.
